# The role of FDG PET/CT or PET/MRI in assessing response to neoadjuvant therapy for patients with borderline or resectable pancreatic cancer: a systematic literature review

**DOI:** 10.1007/s12149-021-01629-0

**Published:** 2021-05-28

**Authors:** Laura Evangelista, Pietro Zucchetta, Lucia Moletta, Simone Serafini, Gianluca Cassarino, Nicola Pegoraro, Francesca Bergamo, Cosimo Sperti, Diego Cecchin

**Affiliations:** 1 Nuclear Medicine Unit, Department of Medicine DIMED, University of Padua, Via Giustiniani 2, 35128 Padua, Italy; 2 Department of Surgery, Oncology and Gastroenterology-DiSCOG, University of Padua, Via Giustiniani 2, 35128 Padua, Italy; 3 Medical Oncology Unit 1, Veneto Institute of Oncology IOV-IRCCS, Via Gattamelata 64, 35128 Padua, Italy

**Keywords:** Pancreatic cancer, FDG, Neoadjuvant therapy, PET/CT, PET/MRI

## Abstract

The aim of the present systematic review is to examine the role of fluorodeoxyglucose (FDG) positron emission tomography (PET) associated with computed tomography (CT) or magnetic resonance imaging (MRI) in assessing response to preoperative chemotherapy or chemoradiotherapy (CRT) for patients with borderline and resectable pancreatic ductal adenocarcinoma (PDAC). Three researchers ran a database query in PubMed, Web of Science and EMBASE. The total number of patients considered was 488. The most often used parameters of response to therapy were the reductions in the maximum standardized uptake value (SUV_max_) or the peak standardized uptake lean mass (SUL_peak_). Patients whose SUVs were higher at the baseline (before CRT) were associated with a better response to therapy and a better overall survival. SUVs remaining high after neoadjuvant therapy correlated with a poor prognosis. Available data indicate that FDG PET/CT or PET/MRI can be useful for predicting and assessing response to CRT in patients with resectable or borderline PDAC.

## Introduction

Pancreatic ductal adenocarcinoma (PDAC) carries a poor prognosis. Survival rates are very low, ranging from 20 to 5% after 1–5 years for all stages combined [1]. The proportion of PDAC resectable at the time of diagnosis is approximately 20–30% [2]. In cases of locally advanced cancer with borderline resectability, stereotactic body radiotherapy and concurrent chemotherapy might increase the chances of curative surgery, thereby improving prognosis [3]. Assessing response to therapy is sometimes difficult with the usual morphological imaging modalities such as computed tomography (CT) and magnetic resonance imaging (MRI) because they are unable to distinguish persistent tumor from postoperative or post-radiation changes [4]. Positron emission tomography (PET) with 18F-fluorodeoxyglucose (FDG) can be used to measure the tumor’s metabolic rate before and after treatment, contributing to an assessment of the efficacy of combined therapies. Hybrid PET/CT scanners have already been employed to measure response to neoadjuvant therapy in a number of solid tumors [5]. Some experiences are now available for PDAC too [6]. The aim of the present systematic review is to investigate the role of FDG PET/CT or PET/MRI in assessing response to preoperative chemotherapy or chemoradiotherapy (CRT) in patients with borderline and resectable PDAC.

## Materials and methods

### Literature search

A systematic review was conducted in accordance with the preferred reporting items for systematic reviews and meta-analyses guidelines (PRISMA). Three researchers (L.E., G.C., N.P.) ran queries in the PubMed, Web of Science and EMBASE databases to retrieve prospective or retrospective studies on the use of FDG PET/CT or PET/MRI for assessing response to therapy in patients with borderline and resectable PDAC.

The following search strings were used “FDG” AND “PET” AND “pancreatic cancer”, “FDG PET/CT” AND “pancreatic cancer”, “PET” AND “pancreatic cancer”, “PET/CT” AND “pancreatic cancer”, “PET” AND “pancreatic cancer” and “neoadjuvant”, “FDG PET” AND “pancreatic cancer” and “neoadjuvant”, “PET” AND “FDG” AND “pancreatic cancer” AND “response to therapy”, and “FDG PET/CT” AND “pancreatic cancer” AND “response to therapy”. No date limits or language restriction were applied. The literature search was up to date as of 1 January 2021. After excluding duplicates, case reports, case series and review articles, the titles and abstracts of the records retrieved were carefully examined. Full texts of the selected articles were obtained, and those written in the English language were carefully analyzed. The following criteria were used to select the studies of interest: (1) FDG was used as a radiopharmaceutical agent, (2) more than 10 patients were enrolled, (3) patients with resectable PDAC were involved, (4) hybrid imaging (PET/CT or PET/MRI) was used for diagnostic purposes, and (5) baseline and post-treatment PET/CT scans were available. The references in the articles selected were also screened for additional studies.

### Data extraction

The following data were extracted for each article: authors, year of publication, study design, number of patients enrolled, type of neoadjuvant therapy regimen, number of PET/CT or PET/MRI scans, intervals between PET scans and therapy, and details of the image acquisition protocol and the criteria used to assess response to neoadjuvant therapy.

### Quality assessment of studies

The methodological quality of the studies was judged by two investigators (L.E and G.C.) using the “Quality Assessment of Diagnostic Accuracy Studies” tool, v. 2 (QUADAS-2) [7].

## Results

**Fig. 1:**
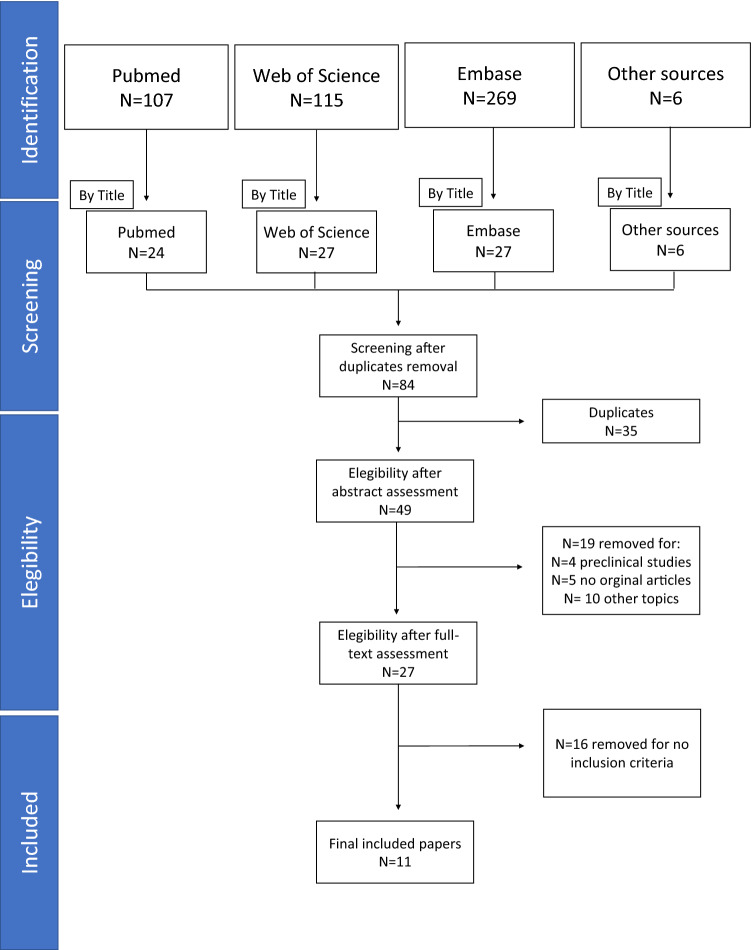
PRISMA diagram for the selection of papers

From a total of 84 papers identified in the databases, 49 studies were eligible after excluding any duplicates. Then further selection led to the retrieval of 27 full texts, but only 11 studies met our inclusion criteria for the present review (Fig. 1).

**Fig. 2:**
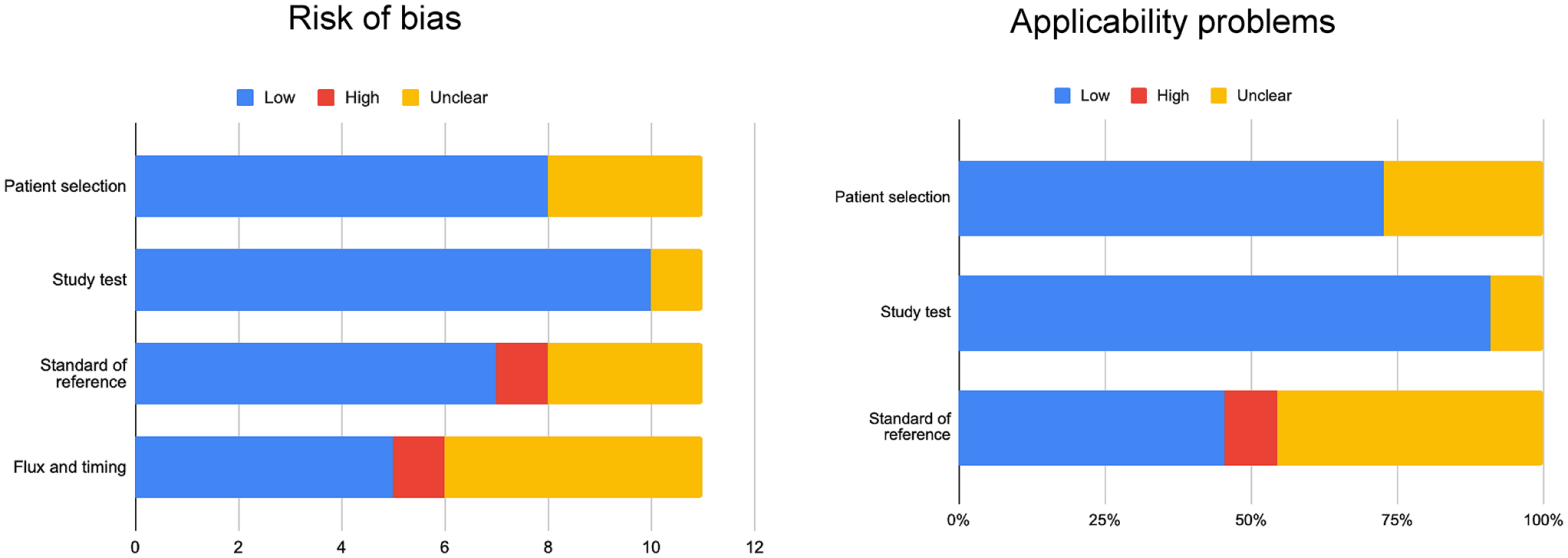
QUADAS-2 results

Based on the QUADAS-2, most of these papers have a low risk of bias and applicability issues. A few papers revealed some unclear data concerning the reference standards used and patient selection, as shown in Fig. 2.

Table 1 lists the characteristics of the studies selected [8–18]. The total number of patients enrolled was 488. Four of the 11 studies (36%) were prospective. In all cases, at least 2 PET/CT or PET/MRI scans had been obtained, one before and one after therapy [8–14, 16–18]. Zimmerman et al. [15] reported on 3 PET/CT scans obtained before, during and after CRT. In 6 studies, a combined therapy was used to treat borderline or resectable PDAC [8, 11, 12, 14–16, 18]. PET/MRI was only used in one study [18].

**Table 1:** Characteristics of selected papers

No.	Author, ref	Year of pub	Country	Study design	N of pts	Chemotherapy (alone or + RT)	Chemotherapy alone	CHT and RT	Type of scanner	N of PET scans	Time among PET scan	Outcome
1	Kittaka et al. [8]	2013	Japan	R	40	Gemcitabine-based chemoradiotherapy	No	Yes	PET/CT	2	Baseline and at least 8 weeks after the completion of radiation therapy	SUV measurement by FDG PET/CT can be a useful tool to select suitable candidates for preoperative CRT and subsequent resection, predicting the locoregional effect of preoperative CRT
2	Ielpo et al. [9]	2016	Spain	P	25	Gemcitabine and nab-paclitaxel	Yes	No	PET/CT	2	Baseline and preoperative scan	SUV from FDG PET can help in defining the response to therapy
3	Mellon et al. [10]	2017	USA	R	70	Gemcitabine or GTX or folfirinox or gem + abraxane or others	Yes	No	PET/CT	2	Baseline Post-CRT	Pre-operative PET/CT and CA19.9 response correlate with histopathologic tumor regression
4	Akita et al. [11]	2017	Japan	R	83	Gemcitabine-based chemoradiotherapy	No	Yes	PET/CT	2	Baseline and at least 8 weeks after the completion of chemoradiation	FDG PET/CT is useful to evaluate the efficacy of preoperative therapy for PDAC
5	Sakane et al. [12]	2017	Japan	R	25	Gemcitabine-based chemoradiotherapy	No	Yes	PET/CT	2	Baseline after the completion of therapy	Higher post-treatment SUL_peak_ and positive MTV/TLG could predict the unfavorable histopathological effects of CRT in patients with pancreatic adenocarcinoma
6	Dalah et al. [13]	2018	USA	P	15	Gemcitabine or xeloda and folfirinox or gemcitabine and abraxane	Yes	No	PET/CT	2	Baseline after 3–7 weeks from the end of chemotherapy	FDG PET can be more informative than CT for the definition of response to therapy
7	Barnes et al. [14]	2020	USA	R	104	Gemcitabine-based chemoradiation or capecitabine-based chemoradiotherapy	No	Yes	PET/CT	2	Baseline and approximately 4 weeks following the completion of neoadjuvant therapy	CA19.9 monitoring mirrors quantitative changes in the burden of disease, SUV_max_ levels may provide complimentary information in estimating the tumor’s biologic behavior
8	Zimmermann et al. [15]	2020	Germany	P	18	Gemcitabine and oxaliplatin radiochemotherapy	No	Yes	PET/CT	3	Baseline PET after two courses of NAT after 10 weeks from NAT (chemo + RT)	FDG PET/CT may be a reliable method to evaluate response to the combined therapy
9	Yokose et al. [16]	2020	Japan	R	22	Gemcitabine and nab-paclitaxel or TS1 + cisplatin + mitomycin and radiotherapy	No	Yes	PET/CT	2	Baseline and 2–3 weeks after completion of neoadjuvant treatment	PERCIST more accurately reflected neoadjuvant treatment’s therapeutic effect on PDAC than RECIST
10	Barbour et al. [17]	2020	Australia	P	42	Gemcitabine and nab-gemcitabine	Yes	No	PET/CT	2	Baseline and after 15 days from the start of therapy	PET/CT cannot be able to detect an early response to nab-gem in patients with advanced pancreatic cancer
11	Panda et al. [18]	2020	USA	R	44	Gemcitabine and oxaliplatin radiochemotherapy	No	Yes	PET/MRI	2	Baseline and post-NAT	Metabolic metrics from PET/MRI and morphological metrics from CT may help assess pathologic response to NAT as well as predict survival. CA 19.9 does not correlate with the outcome

*CHT +*  chemotherapy, *RT* radiotherapy, *R* retrospective, *P* prospective, *CRT* chemoradiation therapy, *PDAC* pancreatic adenocarcinoma, *SUL* standardized uptake lean, *MTV* metabolic tumor volume, *TLG* total lesion glycolysis

Table 2 shows methodological details for the studies considered. The most often used parameters of response to therapy were the reduction in the maximum standardized uptake value (SUV) or the peak standardized uptake lean mass (SUL) [8–16, 18]. In particular, reductions in SUV_max_ [or the regression index (RI)] of more than 50% were associated with a more favorable response to therapy than in the case of smaller reductions [8, 10, 11, 14, 15, 18]. In two studies, changes in metabolic volume parameters or other specific criteria (i.e., PET response criteria in solid tumors—PERCIST or SUV_max_ ×  blood glucose level/100−SUV_gluc_) were also used to assess response to neoadjuvant therapy [13, 16], demonstrating the additional value of alternative PET metrics in predicting and assessing response to therapy.

**Table 2:** Imaging PET protocol and interpretation in all studies

No.	Author	No of pts	Time between administration and acquisition	Acquisition duration	Glycemia (mg/dL)	Administered FDG dose	PET criteria for the assessment of response to NAT	SUV_max_ median reduction (%)
1	Kittakaet al [8]	40	120 min	NA	NA	3.7 MBq/kg (mean dose 200 Mbq)	SUV_max_ reduction	53.0 ± 19.0 in responder 41.0 ± 12.0 in non-responder
2	Ielpo et al. [9]	25	NA	NA	NA	NA	SUV reduction	41.8 (SUV_mean_ 7.9 pre-neoadjuvant 4.6 post-neoadjuvant)
3	Mellon et al. [10]	70	> 90 min	NA	< 200	NA	SUV_max_ reduction	61.1
4	Akita et al. [11]	83	120 min	NA	104 ± 29.7	3.7 MBq/kg (mean dose 200 Mbq)	SUV_max_ reduction	44.1 ± 20.3 in poor responder 67.1 ± 15.1 in good responder
5	Sakane et al. [12]	25	60 min	2 min scan/bed position × 11 positions	72–148	3.7 MBq/kg	SUL_peak_, SUV_max_, MTV, TLG	24.0–57.0 in responder 28.0–47.0 in non-responder
6	Dalah et al. [13]	15	45–60 min	NA	NA	10–19 mCi	PERCIST, SUL_peak_	PERCIST
7	Barnes et al. [14]	104	60 min	NA	< 200	Standard dose of 370 mBq for patients weighing < 55 kg, 444 mBq for patients weighing 55–91 kg, and 518 mBq for patients weighing > 91 kg	SUV_max_ reduction	44.0–53.0
8	Zimmermann et al. [15]	18	60 min	NA	NA	5 MBq/kg	SUV_max_ reduction	54.0 (median SUV_max_ decreased from 8.29 baseline to 3.83 at the end of treatment)
9	Yokose et al. [16]	22	60–75 min	120 s for each bed position in the three-dimensional mode	< 200	4 MBq/kg	PERCIST, SUL_peak_, SUV_max_, MTV, TLG	38.8
10	Barbour et al. [17]	42	NA	NA	NA	NA	NA	No level of reduction in SUV_max_ from baseline was predictive of early response to therapy
11	Panda et al. [18]	44	60 min	60 min	< 200	10 mCi	SUV_max_ reduction and SUV_gluc_ reduction	64% change in SUV_max_ 64% change in SUV_gluc_

*NAT* neoadjuvant therapy, *MTV* metabolic tumor volume, *TLG* total lesion glycolysis, *NA* not available, *SUV*  standardized uptake value, *SUL* standardized uptake lean

Some authors wrote that patients with higher baseline (pre-CRT) SUVs were associated with a better response to therapy [8], and a better overall survival (OS) [14]. SUVs remaining high after neoadjuvant therapy correlated with a poor prognosis [12]. In addition to the absolute SUVs before and after therapy, many authors found the RI useful for assessing response to therapy [8, 10, 11] for the purposes of selecting candidates suitable for subsequent resections. Associating changes in SUV_max_ with other PET measurements—such as metabolic tumor volume (MTV), total lesion glycolysis (TLG) or SUL_peak_—could improve the prediction of response to treatment and OS [18]. Barnes et al. [14] found that PET data in association with carbohydrate antigen 19–9 (CA 19–9) levels could significantly predict outcome and response to CRT, though Panda et al. [18] reported that CA 19–9 levels showed some limitations in predicting OS.

In contrast with the above-mentioned findings, Barbour et al. [17] reported that SUV_max_ did not predict outcome or response to therapy, and Zimmerman et al. [15] only found a trend toward a predictive value for response to neoadjuvant therapy on FDG PET. That said, Barbour et al. [17] had acquired PET images very soon (only 15 days) after starting neoadjuvant CRT, and Zimmerman et al. [15] had enrolled only a very limited number of patients (*n* = 15) in the context of a clinical trial.

Dalah et al. [13] found that 15% of patients had progressive disease when using PERCIST criteria, as opposed to only 7% when they applied response evaluation criteria in solid tumors version 1.1 (RECIST1.1). In other words, PERCIST appears to increase the chances of detecting patients with progressive disease. Yokose et al. [16] also aimed to confirm the usefulness of PERCIST for assessing the effect of treatment based on the pathological response to treatment, and to examine the prognostic utility of FDG PET/CT parameters. They confirmed that PERCIST and SUV_max_ were superior to RECIST for the purpose of assessing the effects of preoperative treatment.

Zimmermann et al. [15] examined the prognostic value of FDG PET and diffusion-weighted MRI (DWI) obtained before and then twice during neoadjuvant treatment. FDG PET/CT identified responders and non-responders more accurately than the mean apparent diffusion coefficient (ADC). Panda et al. [18] also showed that hybrid PET/MRI can help ascertain pathological response to therapy in PDAC with a high negative predictive value.

Table 3 summarizes the major findings in terms of the surgical outcomes and the consistency between findings on histopathology and imaging. The complete resection (R0) rate ranged between 64 and 100% of the population studied. The rate of responders to neoadjuvant therapy exceeded 50% in 5 studies [8, 9, 11, 16, 17]. Based on a semiquantitative score (the RI), the consistency between histopathology and FDG PET data was reportedly in the ranges of 67–93% for responders, and 53–74% for non-responders.

**Table 3:** Data about surgery, histology and PET imaging in all studies

No.	Author	Surgical treatment	N of R_0_	Histological evaluation	PET responders	Agreement between PET findings and histology
1	Kittaka et al. [8]	NA	40/40 (100%)	Response = 21 (53%) No response = 19 (47%)	RI < 46% = 20 RI > 46% = 20	Responders 71% No responders = 74%
2	Ielpo et al. [9]	17/25 PD = 10 DP = 4 TP = 3	17/17 (100%)	CR or near CR = 13 (76%) PR = 2 (11%) No response = 3 (13%)	NA	NA
3	Mellon et al. [10]	NA	NA	CR or near CR = 34 (42%) PR = 37 (46%) No response = 10 (12%)	NA	NA
4	Akita et al. [11]	PD = 47 DP = 34 TP = 2	83/83 (100%)	Poor response 69 (83%) Good response = 14 (17%)	RI < 50% = 44 RI > 50% = 39	Responders = 13/14 (93%) No responders = 43/39 (62.3%)
5	Sakane et al. [12]	NA	NA	CR = 0 PR or near PR = 17 (68%) No response = 8 (32%)	SUL_peak_ reduction < 41.3 = 13 SUL_peak_ reduction > 41.3 = 12	Responders = 4/6 (67%) No responders = 10/19 (53%)
6	Dalah et al. [13]	NA	NA	NA	NA	NA
7	Barnes et al. [14]	148/201	126/1 48 (85%)	CR or near CR = 27/148 (18%) PR or near PR = 121/148 (82%)	NA	NA
8	Zimmermann et al. [15]	In 16 pts PD = 7 DP = 7 TP = 2	12/16 (75%)	NA	RI > 30% = 15/16 RI < 30% = 1/16	Responders = 85% No responders = 58.3%
9	Yokose et al. [16]	NA	14/22 (64%)	Response = 16 (67%) No response = 8 (33%)	NA	Responders = 9/12 (75%) No responders = 5/8 (63%)
10	Barbour et al. [17]	NA	25/29 (86%)	Response = 15 (52%) No response = 14 (48%)	NA	NA
11	Panda et al. [18]	PD = 29 DP = 7 TP = 8	44/44 (100%)	CR or near CR = 19/44 (43%) PR or near PR = 25/44 (57%)	NA	NA

R_0_ clear margins, *NA* not available, *RI* retention index, *PD* pancreatoduodenectomy, *DP* distal pancreatectomy, *TP* total pancreatectomy, *CR* complete response, *PR* partial response

## Discussion

The present systematic review raises some points worth discussing. First, a high SUV_max_ on baseline PET scans in patients with resectable or borderline PDAC is associated with a better response to neoadjuvant CRT. Although it is often associated with a more aggressive disease than in the case of tumors with a lower SUV, a rapid cancer cell proliferation rate is associated with a greater chemosensitivity and radiosensitivity. The choice of an appropriate treatment regimen depends on tumor stage, and also on certain other biological and genetic predictors, and a high baseline SUV in patients with resectable or borderline primary PDAC could be seen as an additional predictor of responsiveness and survival.

Second, cancers usually form an inhomogeneous mass with interstitial fibrosis in which the underlying structure is replaced by fibrous tissue containing a number of tumor cells [19, 20]. CRT may exacerbate their inhomogeneity as a result of its cytopathic effect, and because coagulation and or necrosis depend on blood flow and oxygenation [21]. Given these histologically diverse settings, semiquantitative PET parameters reflect the whole-lesion viability of PDAC. In a recent meta-analysis, Wang et al. [8] showed that patients with a greater reduction in SUV_max_ after different kinds of adjuvant treatment tended to have better survival rates (HR 0.68, 95% CI, 0.47–0.98, *p* = 0.037). The authors did not differentiate between neoadjuvant and adjuvant therapy; however, a careful analysis of the findings of the present review shows that a reduction in SUV in the range of 40–60% can be considered as the best cutoff for classifying response to neoadjuvant CRT in patients with resectable or borderline PDAC (see Table 2). The association between reductions in MTV and TLG can further reinforce the final assessment of the efficacy of therapy, and correlation with CA 19–9 levels seems too. Little evidence is available as yet, however, on the prognostic value of replacing reductions in SUVs with PERCIST criteria in this disease setting. Patients with favorable SUVs but less favorable RECIST criteria after preoperative treatment would proceed to resection in the hope of benefiting from surgery. Indeed, a significant reduction in SUVs after CRT was also associated with a better prognosis after a median 40–44 months of follow-up [11, 14]. On the other hand, a patient with an incomplete metabolic response or limited reduction in SUV_max_ or SUV_gluc_ on post-treatment metabolic imaging is unlikely to achieve a complete histopathological response after surgery. These patients might benefit from further chemotherapy or a more aggressive treatment before undergoing resection (rather than immediate radical surgery) to prevent early metastatic spread. As emerged from a careful analysis of Table 3, however, many patients with a lower RI (i.e., metabolic non-responders) showed a less than 80% agreement between their RI and their histopathological findings. RI alone probably cannot be a valuable surrogate parameter for assessing response to neoadjuvant therapy in borderline resectable and locally advanced PDAC, so its association with other biological parameters is essential.

On a third point, although dynamic changes in SUV after preoperative therapy can be useful for predicting the pathological response and prognostic benefit of surgery, there are some issues with the use of FDG PET for assessing the efficacy of neoadjuvant treatment. One major problem concerns clinical conditions other than cancer that influence the SUV. For instance, radiation therapy occasionally causes acute inflammatory changes in surrounding non-cancerous tissues, giving rise to false-positive cases mainly because these inflammatory changes and the presence of metabolically active leukocytes and macrophages lead to erroneously high SUVs. The timing of FDG PET/CT after treatment is consequently important. In the present review, 3 studies conducted repeat (post-CRT) FDG PET/CT or PET/MRI scans less than 4 weeks after the first [14, 16, 17], while 4 studies did so more than 4 weeks after the end of CRT [8, 11, 13, 15]. Such a variability in the present literature makes it hard to say for sure, but it would seem to be best to wait at least 6 weeks after CRT before obtaining a follow-up scan. The proposal of 6 weeks can be summarized as follow. Usually, the time from the end of chemotherapy or CRT and surgery is 6 weeks, as reported in some of the selected papers [9, 15, 17]. The opportunity to make a FDG PET/CT or PET/MR scan very close to the surgical approach, after neoadjuvant therapy, would be useful. Moreover, a median time between 4 and 8 weeks would be considered enough for reducing the inflammatory processes in the surrounding tumor tissues after completion of RT.

Blood sugar levels before and after therapy may vary, particularly in patients with a low insulin production, and this can further affect SUVs.

Finally, little information is available as yet on the utility of PET/MRI for assessing response to therapy in resectable or borderline PDAC. The only published paper [18] discusses the PET metric data rather than the advantages in terms of contrast resolution offered by MRI in the integrated scanner. The role of DWI and ADC is still controversial in this setting of patients [13]. Hybrid PET/MRI scanners could facilitate the assessment of the R_0_ resection rate with a view to improving the complete response rate after surgery, and the OS as a consequence. Prospective trials are needed in this setting, however.

In conclusion, the available data show that PET/CT or PET/MRI with FDG have potential as tools for predicting and assessing response to CRT in patients with resectable or borderline PDAC. They can also be useful for the prognostic stratification of patients after CRT. That said, the small numbers of patients enrolled in each study, the different criteria used to assess response to therapy, and the diverse therapy regimens all go to show that more efforts are needed to conduct well-designed prospective trials.
